# The Effect of Training Experience on Cardiac Morphology in Resistance Exercise Practitioners: A Study on Left Ventricular Systolic and Diastolic Parameters and Left Atrium Mechanical Functions

**DOI:** 10.3390/medicina60122008

**Published:** 2024-12-04

**Authors:** Ahmet Kurtoğlu, Ertuğrul Kurtoğlu, Bekir Çar, Özgür Eken, Jarosław Muracki, Edi Setiawan, Madawi H. Alotaibi, Safaa M. Elkholi

**Affiliations:** 1 Department of Coaching Education, Faculty of Sport Science, Bandirma Onyedi Eylul University, 44170 Balikesir, Türkiye; 2 Department of Cardiology, Medical Faculty, Malatya Turgut Ozal University, 44170 Malatya, Türkiye; 3 Department of Physical Education and Sport Teaching, Faculty of Sport Sciences, Bandirma Onyedi Eylul University, 44170 Bandirma, Türkiye; 4 Department of Physical Education and Sport Teaching, Faculty of Sport Sciences, Inonu University, 44170 Malatya, Türkiye; 5 Department of Physical Culture and Health, Institute of Physical Culture Sciences, University of Szczecin, 70-453 Szczecin, Poland; 6 Faculty of Teacher Training and Education, Universitas Suryakancana, Cianjur 43211, Indonesia; 7 Department of Rehabilitation Sciences, College of Health and Rehabilitation Sciences, Princess Nourah bint Abdulrahman University, P.O. Box 84428, Riyadh 11671, Saudi Arabia

**Keywords:** sport experience, left atrium, left ventricle, mechanical function

## Abstract

*Background and Objectives*: Resistance exercises (REs) are a type of physical activity that individuals from many age groups have been doing recreationally, both as amateurs and professionally, in their daily lives in recent years. It is crucial to understand the effects of such sports on cardiac morphology in order to maximize the benefit of training and to tailor the training content accordingly. The aim of this study was to investigate the relationship between training experience (TE) and left ventricular (LV) systolic and diastolic parameters and left atrial (LA) mechanical function in healthy subjects who regularly performed RE for different durations. *Materials and Methods*: Forty-five healthy adults [age = 28.91 ± 10.30 years, height = 178.37 ± 5.49 cm, weight = 83.15 ± 13.91 kg, body mass index = 26.03 ± 3.42 kg/m^2^, TE = 7.28 ± 6.49 years] who performed RE between 1 year and 20 years were included in our study. The transthoracic echocardiograms (ECHOs) of the participants were evaluated by the cross-sectional research method, which is often used to understand the current situation in a given time period. Correlations between TE and LV systolic and diastolic parameters and LA mechanical function were analyzed. *Results*: As a result, interventricular septal thickness (IVS; r = 0.33, *p* = 0.028), the aortic diameter systole (ADs; r = 0.56, *p* < 0.001), and aortic diameter diastole (ADd; r = 0.58, *p* < 0.001) were positively correlated with TE, indicating associations with increased left ventricular (LV) hypertrophy and reduced ventricular compliance, while the aortic strain (AS; r = −0.44, *p* = 0.002), aortic distensibility (AD; r = −0.62, *p* < 0.001), and diastolic flow parameters including E (r = −0.41, *p* = 0.005), E/A (r = −0.38, *p* = 0.011), and E/Em (r = −0.31, *p* = 0.041) were negatively correlated with TE, reflecting impairments in diastolic function. *Conclusions*: This study showed that diastolic parameters were adversely affected in chronic RE. Therefore, we think that these individuals may have decreased relaxation and filling functions of the heart, which may also reduce adequate oxygen and nutrient delivery to the tissues. In this context, cohort studies are needed to analyze in detail the reasons for the decrease in diastolic parameters in these individuals.

## 1. Introduction

In athletes, the cardiac system undergoes some chronic and acute functional changes after different exercise activities [1,2,3]. In particular, cardiac hypertrophy (sports cardiomegaly) occurs, and this hypertrophy includes some changes, such as the enlargement of the cardiac chambers [4], increased wall thickness [5], and increased cardiac output [6]. On the other hand, cardiovascular adaptation also meets the increasing demands of athletes during these high- and low-intensity loads. Especially in sports that require high cardiac load, such as resistance exercise (RE), the effects on the ventricular and atrial structures of the heart may be remarkable [7]. The left ventricle (LV) is one of the most sensitive structures in this adaptation mechanism and tends to improve both diastolic and systolic function to meet the increased oxygen and nutrient demands, especially with exercise [8].

LV systolic and diastolic functions respond differently to different long-term sports disciplines [9]. In endurance sports, the diastolic filling rate and volume of the LV increase, leading to favorable improvements in left ventricular diastolic function [10,11]. RE, on the other hand, generally causes concentric hypertrophy of the LV wall [12,13]. In individuals performing RE, this may lead to the loss of elasticity in the filling phase of the heart and an increased risk of diastolic dysfunction [14]. RE also improves the mechanical function of the left atrium (LA). Some studies have suggested that intense RE may adversely affect the left atrial reservoir, conduction, and contractile functions [15].

The pressure on the heart during RE leads to some changes in cardiac morphology [7]. In particular, the increase in arterial pressure increases the afterload level of the heart and produces some changes in LV systolic performance [16]. This causes some acute and chronic changes in the cardiac structure. For example, during acute RE, the diastolic phase of the heart may be forced due to high intrathoracic pressure [17]. This diastolic strain may eventually lead to some diastolic disturbance in the long term. In addition, the change in systolic parameters due to the loss of elasticity in the myocardium with age also necessitates the examination of changes in systolic parameters. In a study supporting this, Gong et al. argued that the isovolumic relaxation time (IVRT), LA volume (LAV), early diastolic filling (E), and late diastolic contraction (A) were negatively affected with age [18].

REs have an effect on the systolic and diastolic functions of the LV [13], and the LA mechanical functions [7] play an important role in the regulation of LV diastolic function. These atrial mechanical functions include the reservoir, conduction (conduit), and contraction (emptying) phases [19]. When increased intrathoracic pressure during RE increases the afterload of the LV [17], the LA makes significant adaptations in these three main functions [20]. In particular, the LA reservoir process may be forced to expand with RE in order to store more blood [15]. Over time, this may lead to atrial dilatation due to increased pressure and volume overload in the LA and, thus, decreased atrial elasticity. Consequently, these continuous stresses may increase the amount of connective tissue, weakening the structural integrity of atrial myocytes. This may lead to some reduction in the contractile function of the LA.

Sedentary individuals have been known to have some alterations in cardiac function with increasing age [21]. The incidence of alterations in cardiac functions, such as arterial stiffness, myocardial concentric hypertrophy, diastolic dysfunction, decreased ejection fraction, heart valve problems, autonomic nervous system problems, etc., increases with age [22,23,24,25,26]. Although chronic aerobic exercise such as swimming, long-distance running, cycling, etc., effectively minimizes such cardiac problems, we think that it may be of paramount importance to analyze in depth the effects of exercise modalities, including the different content, intensity, and types of aerobic and anaerobic loading conditions on the heart. In the literature, although there are many studies on the effects of RE on cardiac structure, the findings on how systolic and diastolic functions and left atrial mechanical functions are affected by training experience (TE) are limited. Therefore, the aim of our study was to investigate the TE-related change in LV systolic and diastolic parameters and LA mechanical functions in subjects with RE. In this way, we plan to analyze the specific changes in cardiac morphology due to RE in a multidimensional manner. Thus, this study is expected to provide the opportunity to shape the exercise content to provide cardiac remodeling to individuals performing RE. In this context, the hypothesis of our study was determined as ‘there is a relationship between LV systolic and diastolic parameters and LA mechanical function in individuals performing RE for different durations’.

## 2. Methods

This study was designed as a cross-sectional study to evaluate the cardiac morphological changes in individuals who performed RE for different durations [27]. In this context, LV systolic and diastolic parameters and LA mechanical functions were analyzed by this method.

### 2.1. Participants

Between January 2023 and January 2024, male participants attending fitness centers with different equipment and the opportunity to exercise with free weights in the Balıkesir Bandırma district were randomly included in this study. In this context, 45 volunteer male participants aged between 19 and 50 years were included in our study (Table 1). Voluntary participants who attended fitness centers at least 2 days a week for at least 1 year and whose weekly exercise volume was at least 150 min were included in the study. The weekly exercise duration of the participants was calculated according to the daily exercise duration reported by the participants [28]. Participants with diabetes, arrhythmia, valvular heart disease, hypertension, coronary artery disease, thyroid-like problems, autonomic nervous system disease, hospitalized with a cardiac problem in the last 6 months, active infection, using drugs that increase fat burning but do not naturally increase muscle rate, such as beta-androgenic agonists, and using anabolic steroids were not included in the study.

The minimum sample size was calculated using G-Power (version 3.1). Accordingly, exact correlation used the Bivariate normal model; we computed the required sample size given; and α, power and effect size were selected. Accordingly, when α = 0.05, power (1 − β) = 0.80, and effect size = 0.38, it was calculated that there should be at least 39 participants with 80% real power. Although the minimum sample size was 39, 45 participants were invited, and all participants agreed to be included in the study.

The necessary permissions were obtained from the Inonu University Institute of Health Sciences Non-Interventional Ethics Committee for this study (2023/4749). In addition, in a meeting attended by all participants, the procedures to be applied in the study, the purpose of the study, and the basic hypotheses were informed, and their voluntary consent was obtained. In addition, this research was conducted in accordance with the ethical principles determined by the Declaration of Helsinki.

### 2.2. Data Collection Tools

#### 2.2.1. Body Surface Area

Firstly, the demographic characteristics of the participants, such as age, height, weight, body mass index, body mass index, and duration of exercise, were determined, and then the body surface area was determined for the standardization of echocardiographic (ECHO) measurements. After this information was obtained, the BSA of the participants was determined by the following formula [29]:

BSA = Weight (kg) × 0.425 × Height (cm) × 0.725 × 0.00718.

#### 2.2.2. Systolic and Diastolic Blood Pressure and Heart Rate

Participants’ heart rates and systolic–diastolic blood pressures were measured after 9 min of complete passive rest. All measurements were performed with an Erka Brand (Erka Perfect Aneroid/Germany) stethoscope and a sphygmomanometer [30].

#### 2.2.3. ECHO Measurements

All ECHO evaluations were performed by the same cardiologist at the same time of day (10:00–12:00 a.m.) and at room temperature (20–22 °C). Before the measurements, the participants were warned to get eight hours of sleep, not to consume food and beverages other than water from 3 h before the measurements, and not to participate in any physical activity in order to not affect the ECHO results. All ECHO examinations were performed using a Vivid T8 device and a 3ScRS transducer (GE Medical System, Chicago, IL, USA). All measurements were performed according to the recommendations of the American Society of Echocardiography Guidelines [31]. Accordingly, participants were lying in the left lateral decubitus position. The cardiac structure was evaluated in detail from the standard sections of the parasternal long axis, short axis, epical four spaces, two spaces, and subcostal sections. Accordingly, the thickness of the muscular wall (interventricular septal thickness, IVS) separating the right and left ventricles of the heart in the parasternal long axis and the thickness of the LV posterior wall (PWT), which constitutes an important part of the muscle tissue of the heart and reflects the blood pumping capacity of the heart, were measured. Aortic systolic (AD) and diastolic (ADd) diameters were measured from the inner edge of the aortic root. In addition, the LV end-diastolic diameter (LVDD) and end-systolic diameter (LVSD) included the distance between the inner edges of the endocardial borders [31,32].

Left ventricular end-diastolic volume (LVDV), left ventricular end-systolic volume (LVSV), stroke volume (SV), and ejection fraction (EF) were measured by the modified Simpson method in an apical four-chamber view. The apical four-chamber view visualizes the full long axis of the LV, making volume calculations and the assessment of ventricular function possible [33]. Pulsed wave (PW), early diastolic flow velocity (E), late diastolic flow velocity (A), the E/A ratio, ejection time (ET), isovolumic relaxation time (IVRT), isovolumic contraction time (IVCT) and transmissible flow parameters were measured during diastole. Tissue Doppler imaging of the annulus motion was measured at the lateral mitral annulus and peak early systolic (Sm), peak early diastolic (Em), and peak late diastolic (Am) velocities. In addition, each participant performed the Valsalva maneuver to further evaluate all mitral flow parameters. Changes in mitral parameters during this procedure allow for a clearer characterization of diastolic dysfunction [34].

The left ventricular mass index (LVM-I), an important parameter in the evaluation of LV hypertrophy, was calculated using the Devereux formula. In this formula, the three basic anatomical structures IVS, LVDD, and PWT and their parameters evaluated in ECHO are evaluated when the heart is in diastole [35,36]. Aortic strain (AS) and aortic distensibility (AD) were used as aortic elasticity parameters. The following formula was used to calculate these parameters [37]:

$$Aortic\;Strain\;\left(\%\right)=\frac{systolic\;diameter-diastolic\;diameter}{diastolic\;diameter}\times 100$$

$$Aortic\;Distensibility\;\left(10-6.cm-2.dyn-1\right)=\frac{2(Aortic\;Strain)}{systolic\;pressure-diastolic\;pressure}$$

Left atrial volumes were calculated from apical four-chamber and two-chamber images using the dual plane area length method. Maximum left atrial volume (LAVmax) was measured with the mitral valve fully open, minimum left atrial volume (LAVmin) with the mitral valve fully closed, and pre-systolic left atrial volume was measured at the onset of the p wave (LAVp) on the electrocardiogram. All measurements were repeated during three consecutive heartbeats and averaged. The LAVmax index was determined as the maximum volume of the left atrium (LAVmax) divided by the body surface area. All volumes were corrected by dividing by the LAVmax index. Left atrial mechanical function was determined using the following formula [38].

LA passive emptying volume (LAPEV) = LAV max − LAVp

LA passive emptying fraction (LAPEF) = LAPEV/LAVmax.

LA active emptying volume (LAAEV) = LAVp − LAVmin.

LA active emptying fraction (LAAEF) = LAAEV/LAVp.

LA total emptying volume (LATEV) = LAVmax − LAVmin.

LA total emptying fraction (LATEF) = LATEV/LAVmax.

Conduction volume (CV) = Left ventricular stroke volume − (LAVmax − LAVmin).

In our study, another cardiologist performed the same measurements at different times together with the principal investigator for ECHO intraobserver and interobserver variability. The measurements between them showed an average of 97.8% similarity in all parameters.

### 2.3. Statistical Analysis

The statistical analysis of this study was performed with Python 3.12.3 (PSF, Amsterdam, The Netherlands) and SPSS 25 software (IBM, Armonk, NY, USA). The normality of the ECHO data obtained in this study was analyzed by Shapiro–Wilk and Q–Q plot graphs. Levene’s test was used to test the homogeneity of variances. The quantitative data obtained in this context were found to be normally distributed, and descriptive statistics were given as the mean and standard deviation (SD). In this context, the relationship between the participants’ TE and LV systolic and diastolic parameters and left atrial mechanical function was determined by Pearson correlation analysis. This correlation was visualized with a heatmap. Accordingly, correlation coefficients were determined as <0.1 = insignificant; 0.1–0.3 = small; 0.3–0.5 = moderate; 0.5–0.7 = large; 0.7–0.9 = very large; and >0.9 = almost perfect [39]. The significance level in this study was determined as 0.05. The analyses were performed in Python using pandas, scipy, and statsmodels libraries, and the results were supported with matplotlib and seaborn libraries for visualization purposes.

## 3. Results

**Figure 1:**
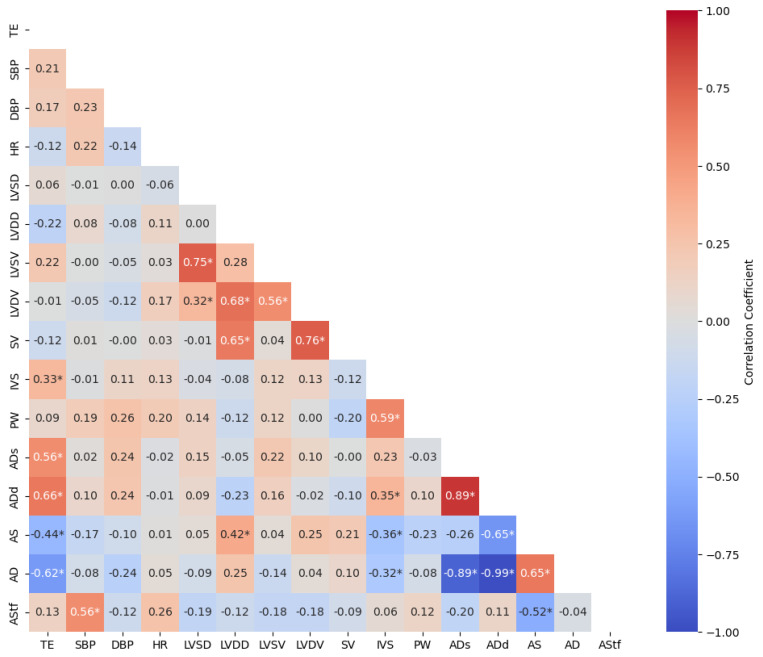
Correlation between participants’ TE and ECHO parameters, SBP, DBP, and HR: TE = training experience; SBP = systolic blood pressure; DBP = diastolic blood pressure; HR = heart rate; LVSD = left ventricular systolic diameter; LVDD = left ventricular diastolic diameter; LVSV = left ventricular systolic volume; LVDV = left ventricular diastolic volume; SV = stroke volume; IVS = interventricular septal thickness; PW = posterior wall thickness; ADs = aortic diameter in systole; ADd = Aortic diameter in diastole; AS = aortic strain; AD = aortic distensibility; AStf = aortic stiffness; I: index by body surface area; * *p* < 0.05.

In Figure 1, the correlation between participants’ TE and ECHO parameters, SBP, DBP, and HR results were analyzed. Accordingly, there was a highly significant positive correlation between the participants’ TE and IVS (r = 0.33, *p* = 0.028), ADs (r = 0.56, *p* < 0.001), ADd (r = 0.66, *p* < 0.001), and a significant negative correlation between the AS (r = −0.44, *p* = 0.002) and AD (r = −0.62, *p* < 0.001). There was no significant correlation between other ECHO parameters and TE.

**Figure 2:**
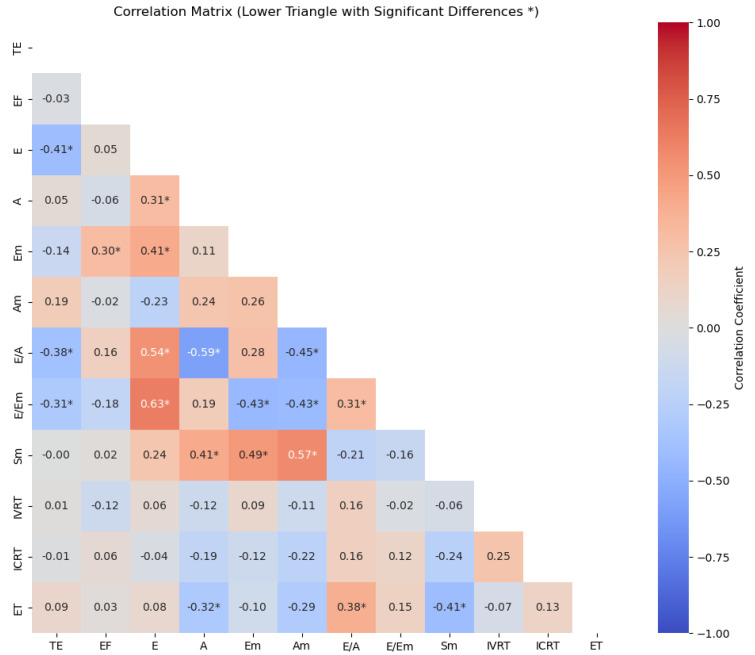
Correlation between left ventricular systolic and diastolic parameters and the TE of participants: TE = training experience; EF = ejection fraction; E = early diastolic flow rate; A = late diastolic flow rate; Em = peak early diastolic; Am = peak late diastolic; Sm = peak early systolic; IVRT = isovolumic relaxation time; ICRT = isovolumic contraction time; ET = ejection time; I: index by body surface area; * *p* < 0.05.

Figure 2 shows the correlation results of the relationship between the participants’ TE and systolic and diastolic parameters. Accordingly, a highly significant negative correlation was found between the TE and E wave (r = −0.41, *p* = 0.005), E/A ratio (r = −0.38, *p* = 0.011), and E/Em (r = −0.31, *p* = 0.041). TE was not associated with other systolic and diastolic parameters (*p* > 0.05).

**Figure 3:**
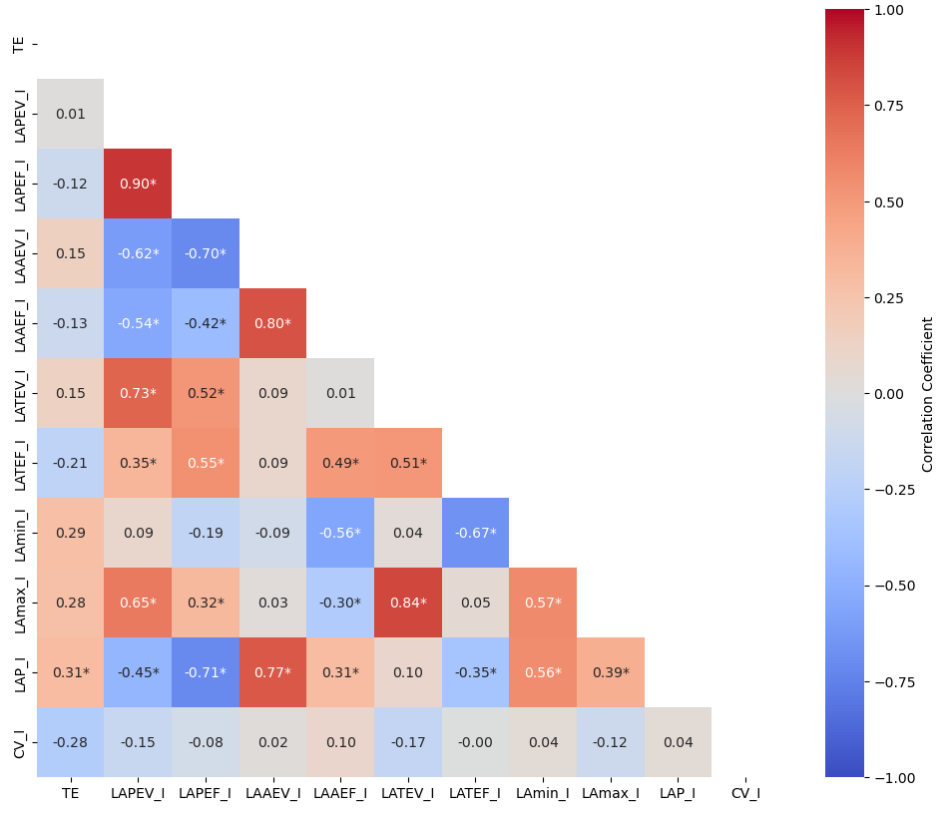
Investigation of the relationship between TE and left atrial mechanical function: TE: training experience; LAPEV: LA passive emptying volume; LAPEF: LA passive emptying fraction; LAAEV: LA active emptying volume; LAAEF: LA active emptying fraction; LATEV: total emptying volume; LATEF: total emptying fraction; LAmin: minimum left atrial volume; LAP: left atrial pressure; CV: conduit volume; I: index by body surface area; * *p* < 0.05.

In Figure 3, the correlation between the participants’ left atrial mechanical function and TE is analyzed. Accordingly, there was a significant positive correlation between the participants’ TE and LAP-I values (r = 0.31, *p* = 0.037). Otherwise, no significant correlation was found between LA mechanical functions and TE (*p* > 0.05).

## 4. Discussion

This study aimed to determine the relationship between TE and ECHO parameters in subjects who performed RE for different durations. The results showed a positive correlation between TE and two-dimensional echocardiographic parameters such as IVS, ADs, and ADd. While AS and ADs decreased significantly with increasing sporting age, the IVS value increased with increasing TE. There was a negative correlation between TE and pulse- and tissue-Doppler ECHO parameters such as the E wave, E/A ratio, and E/Em ratio. When three-dimensional LA mechanical functions were analyzed, there was a positive correlation between LAP-I and TE. However, other LA volumes and fractions were not significantly affected by TE. The remarkable result of our study was that some diastolic echocardiographic findings were correlated with the TE in subjects practicing RE. To the best of our knowledge, this is the first study to evaluate different echocardiographic modalities in depth, such as two-dimensional, three-dimensional, and Doppler echocardiography in individuals performing RE.

Chronic exercise is known to cause many physiological [40], psychological [41], and physical changes [42] in human metabolism. One of these changes is the change in the cardiovascular system [7]. Cardiac changes in athletes are defined as ‘Athlete’s Heart’ in the literature, and ECHO is one of the most important methods for the in-depth examination of the athlete’s heart [43]. There are many hypotheses that examine the chronic effect of different types of exercise on the heart. One of the oldest views is the Morganroth hypothesis. According to the Morganroth hypothesis, it was concluded that there was a significant increase in LVDV, PW, and LV-Mass in long-term endurance athletes. However, while there was no change in LVDV in RE, increases in PW and LV-Mass occurred similarly in endurance athletes. These different patterns were defined as eccentric and concentric LV hypertrophy, and it was concluded that increases in preload during endurance exercise and increases in afterload during RE produced different cardiac changes in these exercise types of LV hypertrophy [44]. In a recent study, Morganrot et al. criticized the view that the pressure load associated with the Valsalva maneuver during RE is similar to stress applied to the heart, such as systemic hypertension and aortic stenosis [45]. Some studies have even argued that LV remodeling in individuals performing RE in different sports disciplines depends on the type of strength exercise performed, the absolute amount of weight lifted, the number of sets and repetitions, the rest time between lifts, and the daily calorie intake [46,47,48,49]. According to the findings of our study, TE is also an important factor in cardiac remodeling. In our study, a positive correlation was found between TE and IVS and ADd and ADs, supporting the view of Morganroth et al. and Haykowsky et al. that LV hypertrophy occurs after RE and endurance training [50,51]. This physiological hypertrophy, especially seen in athletes, contributes to the heart building more muscle mass, causing the heart to increase its stroke volume [7].

One of the important findings of our study is the negative correlation between AS, AD, and TE. In our study, AS and AD decreased with increasing TE. AD basically refers to the change in aortic diameter during a one-unit change in blood pressure and provides information about aortic stiffness [52]. RE causes high pressure in the cardiovascular system through short-term muscle contractions, usually with heavy loads [53]. During such exercises, the blood pressure rises temporarily and exerts pressure on the aortic wall [54,55]. Prolonged exposure to this pressure can cause the aorta to lose its elasticity. Nabati et al. reported that systolic and diastolic blood pressure cause changes in the aortic wall and argued that aortic stiffness and elasticity are correlated with age in healthy individuals. He also concluded that aortic stiffness can increase in individuals with high blood pressure for a long time [56]. Ryffel et al. analyzed the relationship between age and AD results in healthy non-elite runners and concluded that AD decreased with increasing age [57]. Based on these results, we suggest that increases in the maximal and total weights lifted during training and the resulting high blood pressure decrease the AD level in individuals with chronic RE in a TE-dependent manner. This hypothesis was confirmed by the fact that sustained high blood pressure causes collagen deposition in the aortic wall, leading to a decrease in elastin fibers [58].

Considering the LV systolic and diastolic parameters in our study, the E wave, E/A ratio, and E/Em ratio decreased with increased TE. Decreases in the E wave suggest impaired rapid filling of the LV [8]. The E/A ratio represents the ratio of early filling (E) to late filling (A) that occurs during atrial contraction. This reduction may be indicative of diastolic dysfunction and may be associated with the decreased relaxation ability of LV [59]. The fact that the E/Em ratio also shows a negative correlation supports the decrease in LV diastolic functions. The E/Em ratio is used to evaluate the diastolic filling pressure [60]. The decrease in this ratio indicates that ventricular filling pressure is arriving and indicates that the decrease in diastolic functions can continue by exposing the heart to higher filling pressure. Prolonged exposure to RE can lead to increased LV muscle mass and the thickening of the IVS and PW [61]. While these changes may be favorable for performance enhancement and skeletal muscle development during the initial phase of exercise, over time, they may lead to a decrease in the ability of the LV to relax [62]. The findings obtained in our study (increase in IVS and decrease in AD) also support the fact that diastolic dysfunction may develop depending on years in individuals who perform RE. Confirming this view, Caminiti et al. examined the acute effects of different types of eccentric RE on LA function and found that the E/Em ratio increased after all RE interventions [63]. This, together with the favorable effect of acute RE on diastolic parameters, supports our view that increased wall thickness increases wall thickness, and myocardial stiffness may eventually develop diastolic dysfunction.

The LA collects blood before it is pumped into the ventricles and regulates the filling pressure of the heart [64]. LAP-I is an indicator of the pressure level in the LA. The capacity of the LA to expand in the passive phase (LAP-I) directly affects the diastolic function of the LV, and therefore, LAP-I is directly related to LV filling pressure [65]. When LV diastolic function is impaired, the LA mechanical function gives us a lot of important information. In individuals performing RE, LV hypertrophy may be accompanied by a hypertrophic response in the LA. This is the response of the LA to the increased pressure demands associated with RE, and the increase in LAP-I reflects the LA’s attempt to compensate for filling pressure and maintain cardiac performance [7]. However, over time, the dilatation of the LA may become pathological. This may later lead to rhythm disturbances such as atrial fibrillation or diastolic dysfunction, resulting in poor ventricular filling [66]. When analyzed in this context, in our study, the LAP-I value increased as the TE increased. The negative correlation of the E wave, E/A ratio, and E/Em ratio in systolic and diastolic parameters with the age of sport may be an indicator of diastolic dysfunction [59]. The decrease in diastolic filling velocity parameters with age and the increase in LAP-I indicate that the ventricle requires a more atrial contribution during the filling phase [65]. In this situation, the LA has to work harder to support the LV. Since the decrease in E/Em indicates that ventricular filling becomes more difficult and the pressure increases, the findings we obtained in LA mechanical functions and LV diastolic parameters support each other, and we can conclude that there is first-degree mild diastolic dysfunction in individuals who perform REs as the time spent in this sport increases [67].

### Limitations of the Study

This study has some limitations. One of the most important limitations of our study is that the ECHO parameters of the participants were analyzed cross-sectionally. In future studies, chronic changes in cardiac morphology will be analyzed using a cohort study method. In addition, the exercise status of the participants and the number of years of exercise were obtained from their verbal statements. With some scales to be used, data such as weekly exercise duration, whether they actively compete in different sports branches, etc., can be analyzed using more reliable and valid methods. The sample of this study consists of participants who undertake amateur sports in fitness centers. We think that there is a need for further research that will include different levels of RE and participants who have been guided by an exercise specialist.

## 5. Conclusions

In this study, we investigated the relationship between TE and LV diastolic parameters and the LA mechanical function in patients undergoing RE. It was observed that prolonged exposure to RE adversely affected some diastolic ECHO parameters. Some decreases were observed, especially in diastolic parameters with TE. To favor ventricular filling, the increased pressure in the LA with sporting age may be indicative of deteriorations in diastolic function. The decrease in LV diastolic parameters (E, E/A, E/Em) indicates a decrease in the relaxation ability of the heart, and the negative change in AS and AD may support the deterioration in diastolic function. These findings suggest that the heart may cause changes in cardiac structure with exercise in the long term. Long-term effects of high-pressure exercise such as RE may result in diastolic dysfunction and ventricular stiffness. Therefore, we think that the findings obtained from long-term follow-up studies may provide important results. Lastly, this study underlines the use of not only one echocardiographic modality, such as two-dimensional and/or Doppler echocardiography, but also advanced modalities, such as three-dimensional echocardiography, in sports studies to detect subtle changes in cardiac morphology and function.

## Figures and Tables

**Table 1:** Baseline demographic characteristics of the participants.

Parameters	Mean ± SD	%25 Quartile	%75 Quartile
Age (year)	29.91 ± 10.30	21.0	36.50
Height (cm)	178.37 ± 5.49	175.0	181.50
Weight (kg)	83.15 ± 13.91	73.0	91.50
BMI (kg/m^2^)	26.03 ± 3.42	23.55	27.77
TE (year)	7.28 ± 6.49	1.25	12.50
SBP (mmHg)	124.17 ± 10.35	120.0	130.0
DBP (mmHg)	75.60 ± 9.48	70.0	81.0
HR (beat)	79.04 ± 9.34	72.0	85.50

BMI: body mass index, TE: training experience, SBP: systolic blood pressure, DBP: diastolic blood pressure, HR: heart rate.

## Data Availability

Data supporting the findings of this study are available from the corresponding author upon reasonable request.
